# Gonadal Dysfunction in Wolfram Syndrome: A Prospective Study

**DOI:** 10.3390/diagnostics15131594

**Published:** 2025-06-24

**Authors:** Gema Esteban-Bueno, Juan Luis Fernández-Martínez

**Affiliations:** 1 Clinical Management Unit Almería Periférica, Almería Health District, Andalusian Health Service, 04009 Almería, Spain; 2 Spanish Association for Research and Help to Wolfram Syndrome, 04120 Almería, Spain; 3 Group of Inverse, Optimization and Machine Learning Problems, University of Oviedo, 33007 Oviedo, Spain; jlfm@uniovi.es

**Keywords:** wolfram syndrome, gonadal failure, hypogonadism, DIDMOAD, quality of life

## Abstract

**Background:** Wolfram syndrome (WFS), also known as DIDMOAD, is a rare monogenic neurodegenerative disorder characterized by four key components: non-autoimmune insulin-dependent diabetes mellitus (DM), optic atrophy, sensorineural hearing loss, and diabetes insipidus. Although it significantly affects quality of life, gonadal dysfunction, particularly hypogonadism, remains underrecognized. **Methods:** In total, 45 patients (25 men, 20 women) with genetically confirmed WFS from a single tertiary-care center were prospectively followed to assess gonadal function. Men underwent hormonal evaluations, semen analysis, imaging tests, and testicular biopsies. In women, data on age at menarche, menstrual irregularities, and age at menopause were recorded. Hormonal analyses, including anti-Müllerian hormone (AMH) levels, and imaging tests were also conducted. **Results:** Hypogonadism was identified in 19 men (76.0%), of whom 17 (68.0%) had hypergonadotropic hypogonadism and 2 (8.0%) had hypogonadotropic hypogonadism. Testicular biopsies showed seminiferous tubule damage, Sertoli cell predominance, and reduced Leydig cells. Azoospermia was observed in 12 patients, whereas others presented with oligozoospermia, teratozoospermia, or asthenozoospermia. Most patients exhibited low testosterone levels along with elevated LH and FSH, suggesting primary testicular failure, except for two cases of hypogonadotropic hypogonadism. Correlations between biomarkers, onset age and severity have been analyzed and provide important insights regarding medical treatment. In women, menstrual irregularities were universal, with 20% experiencing premature menopause. Four patients had low AMH levels, with ovarian atrophy in three and a postmenopausal uterus in two, indicating early hypogonadism risk. **Conclusions:** Gonadal dysfunction is a significant yet overlooked feature of WFS, requiring systematic evaluation during puberty and beyond. Proper management is essential to mitigate metabolic disturbances and psychological impacts, including infertility distress, relationship challenges, and quality of life concerns. Addressing sexual health is crucial as WFS patients live longer and aspire to establish relationships or start families.

## 1. Introduction

Wolfram syndrome (WS), also known as DIDMOAD (Diabetes Insipidus, Diabetes Mellitus, Optic Atrophy, and Deafness), is a rare autosomal recessive neurodegenerative disorder affecting multiple systems. Its prevalence is estimated at 1 in 770,000 individuals in the United Kingdom and approximately 1 in 1 million in Spain [1,2]. WS is characterized by early-onset, non-autoimmune insulin-dependent diabetes mellitus and optic atrophy, with subsequent development of diabetes insipidus and sensorineural hearing loss [3,4,5,6,7]. Gonadal dysfunction, though less studied, may also significantly affect patients’ quality of life and contribute to the broader phenotype of the disease [8].

Recent studies suggest that gonadal dysfunction could serve as a relevant diagnostic criterion for WS, particularly in pediatric and young adult patients, underscoring the need for systematic screening and early intervention [9,10,11]. Hypogonadism in WS may manifest as either hypergonadotropic or hypogonadotropic, affecting both males and females [12,13]. In males, primary testicular dysfunction is the predominant form, associated with low testosterone levels and histological findings such as seminiferous tubule hyalinization and Leydig cell depletion [12,13,14]. Genetic underpinnings of hypogonadism, including congenital hypogonadotropic forms, are increasingly recognized in syndromic presentations like WS [14,15,16].

Hypogonadism in males with WS may be classified as primary (low testosterone with high LH/FSH) or secondary (low testosterone with low or normal LH/FSH) [12,13,14,15,16]. Diagnostic criteria include total testosterone levels below 300 ng/dL (10.4 nmol/L), preferably measured in the morning [15]. When SHBG alterations are suspected, free testosterone should also be evaluated [16]. Clinical symptoms include reduced libido, erectile dysfunction, fatigue, muscle loss, and osteoporosis [16,17]. The differential diagnosis is further supported by LH and FSH levels, although not diagnostic on their own [15]. Recent guidelines emphasize evaluating both functional and organic causes of hypogonadism in rare syndromes [18].

In females, gonadal dysfunction may also be primary or secondary but is harder to detect due to variable hormonal patterns and masking effects of contraceptive use [11,19,20,21,22]. Consequently, ovarian insufficiency in WS may go undetected without specific testing [21,22]. AMH measurement and imaging are emerging tools for assessing ovarian reserve [19,22,23]. Some studies advocate for including gonadal dysfunction as a diagnostic feature of WS, alongside urinary abnormalities [10,23,24].

Reproductive dysfunction in WS can affect metabolic health, bone density, and psychological well-being [7,8,24]. Symptoms such as fatigue, weakness, cognitive impairment, and mood disorders are often related to hypogonadism [7,24]. These findings support early hormonal assessment and timely intervention [7]. Although treatments such as testosterone replacement are described in guidelines [15,18,25], they are often underused in WS. Recent cohort studies confirm the high prevalence and variability of gonadal dysfunction in WFS1-spectrum disorders, highlighting the need for structured evaluation [26,27,28].

This study is prospective, aiming to evaluate gonadal function in a cohort of patients with genetically confirmed WS, with special attention to clinical, biochemical, and sex-specific features, to promote early recognition and improve clinical management.

## 2. Materials and Methods

**Study Design and Participants**: This was a prospective cohort study conducted between 1999 and 2024. A total of 45 patients with genetically confirmed Wolfram syndrome (WS) were enrolled in Spain and Portugal, comprising 25 males and 20 females. At the time of data analysis, 10 patients had died, leaving 35 survivors with a mean age of 26.23 years (SD 9.61). All participants carried pathogenic variants in the WFS1 gene, confirmed by Sanger sequencing (BigDye™ Terminator v3.1 Cycle Sequencing Kit; Applied Biosystems, Thermo Fisher Scientific, Waltham, MA, USA) or, in later cases, next-generation sequencing (NGS) multigene panels run on a MiSeq^®^ platform (Illumina Inc., San Diego, CA, USA) for rare endocrine syndromes. 

**Inclusion and Exclusion Criteria:** Inclusion criteria were: (1) clinical diagnosis of WS, and (2) genetic confirmation of pathogenic or likely pathogenic variants in *WFS1*, classified according to ACMG/AMP guidelines, detected by Sanger sequencing or multigene next-generation sequencing (NGS). Patients who did not meet both criteria were excluded. 

**Clinical and Laboratory Evaluation**: All patients were systematically evaluated for gonadal dysfunction. Since diabetes mellitus and optic atrophy are core diagnostic features of WS, their presence was documented in every patient. Diabetes diagnosis included screening for autoantibodies (ICA, IAA, GADA, IA-2A; Euroimmun AG, Lübeck, Germany) to confirm the non-autoimmune nature of WS-related diabetes. ZNT8 antibodies were not measured, as they were not included in the standard screening protocols during the earlier years of the study.

**Male Evaluation**: In males, hormonal profiling included total testosterone, free testosterone, luteinizing hormone (LH), follicle-stimulating hormone (FSH), inhibin B, sex hormone-binding globulin (SHBG), and albumin. Hormone levels were measured using Immulite^®^ 2000 (Diagnostic Products Corporation, Los Angeles, CA, USA; 1999–2006), ADVIA Centaur^®^ XP (Siemens Healthineers, Erlangen, Germany; 2007–2016) and Cobas^®^ e 801 (Roche Diagnostics GmbH, Mannheim, Germany; 2017–2024), with appropriate calibrations across time periods. Semen analysis followed WHO guidelines and assessed sperm concentration, motility, and morphology. Imaging with testicular ultrasound and/or MRI evaluated structural abnormalities. In selected patients, testicular biopsy was performed for histological assessment of seminiferous tubules, Sertoli, and Leydig cell populations. Diagnostic classification of hypogonadism in male patients was based on testosterone and gonadotropin levels, as summarized in Table 1.

**Female Evaluation:** In females, data collected included age at menarche, menstrual regularity, dysmenorrhea, and age at menopause. Hormonal evaluation included anti-Müllerian hormone (AMH), FSH, LH, and estradiol, performed using Beckman Coulter or Roche platforms, with assay-specific cutoffs. Pelvic ultrasound assessed ovarian volume, atrophy, and uterine morphology.

**Classification of Hypogonadism:** Hypogonadism was defined and classified as either primary (hypergonadotropic) or secondary (hypogonadotropic) based on LH and FSH levels. In males, testicular volume was assessed by orchidometry or ultrasounds. Hormonal cutoffs followed international guidelines for age and sex.

**Data Collection:** Patients were recruited across Spain and Portugal. Evaluations were performed either during scheduled multidisciplinary visits or home visits conducted by the principal investigator.

### Study Design, Compliance with Ethical Standards, and Data Analysis

This study was a quantitative, observational, descriptive, and cross-sectional analysis conducted in genetically confirmed WS patients from Spain, with the inclusion of two additional patients from Portugal. The study was conducted in accordance with the Declaration of Helsinki and approved by the Ethics Committee of the Torrecárdenas University Hospital (code 75/2020), approval date 27 February 2020.

Prior to study inclusion, written informed consent was obtained from all participants or their legal guardians. For participants under 18 years of age or those unable to provide informed consent, consent was obtained from a parent or legal guardian. Confidentiality and data protection were ensured in accordance with Spanish Organic Law 3/2018, of 5 December, on the Protection of Personal Data and Guarantee of Digital Rights (Spain), BOE No. 294, 6 December 2018, 119788–119857.

**Data analysis:** Statistical analyses were performed using Python 3.0 statistical libraries, ensuring robust and reproducible computations. Descriptive statistics were applied to all variables, with continuous variables summarized as means ± standard deviations (SD) for normally distributed data and medians with interquartile ranges for non-normally distributed data. Categorical variables were expressed as frequencies and percentages. Correlation analyses were conducted to assess the relationships between hormonal and metabolic biomarkers, age of onset, and severity, identifying statistically significant differences between groups (*p* < 0.05) and relevant associations.

## 3. Results

Diabetes mellitus was present in all cases except one patient with an autosomal dominant WFS1-related disorder. In those with diabetes, the onset occurred before the age of 16 years. In 100% of cases, the diabetes was non-autoimmune, with negative autoantibodies (ICA, IAA, GADA, IA-2A). Optic atrophy was also diagnosed before age 16 in all patients, as confirmed by an ophthalmologist. The patient with the dominant form of WFS1, aged 13 years, did not show clinical or biochemical signs of hypogonadism at the time of evaluation.

### 3.1. Male Patients

#### 3.1.1. General Statistics

Table 2 shows the statistics of the whole population of male patients. Among the 25 male patients, the average luteinizing hormone (LH) level was 12.40 IU/L (SD, 6.91), the average follicle-stimulating hormone (FSH) level was 18.44 IU/L (SD, 13.50), the average albumin level was 4.21 g/dL (SD, 0.52) and the average Sex Hormone-Binding Globulin (SHBG) level was 59.88 g/dL (SD, 26.95). Mean total testosterone was 9.88 nmol/L (SD, 7.06), and free testosterone averaged 0.135 nmol/L (SD, 0.079).

**Figure 1:**
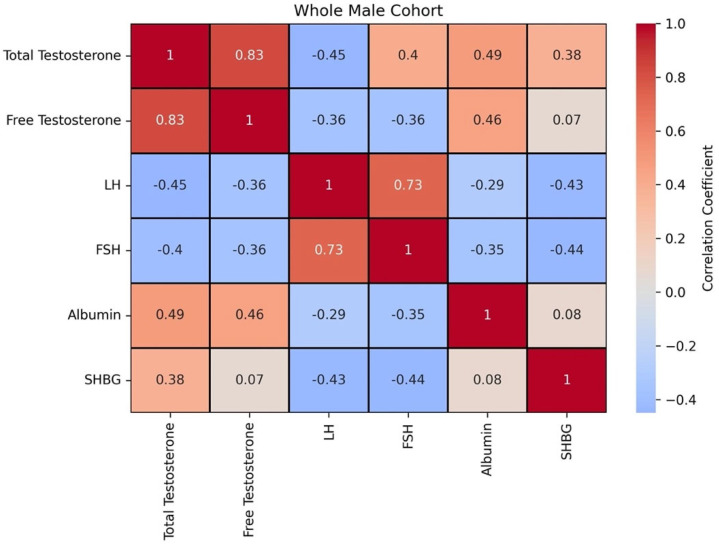
Correlation matrix between biomarkers for the whole male population (correlation coefficient has no units). The highest positive correlation is between total testosterone and free testosterone (0.83), FSH and LH (0.73), and total testosterone and albumin (0.49) and free testosterone and albumin (0.46). Negative weak correlation between FSH and SHBG (−0.44) and LH and SHBG (−0.43).

Figure 1 shows the correlation matrix between these biomarkers for the whole male population. A strong positive correlation can be observed between total testosterone and free testosterone(0.83) and a positive correlation between FSH and LH (0.73). The rest of the biomarkers are weakly correlated (positive or negative) or are uncorrelated.

#### 3.1.2. Males with Hypogonadism

In total, 19 male patients met the criteria for hypogonadism. The mean age of onset was 18.35 years (range, 15–29), with the majority developing symptoms during adolescence. Most cases were classified as severe. The mean total testosterone level was 7.29 nmol/L (range, 2.76–19.06), and the mean free testosterone level was 0.103 nmol/L (range, 0.026–0.239), both indicating consistently low androgen levels. LH values ranged from 1.2 to 25.0 IU/L, with a mean of 13.99 IU/L, reflecting variable hypothalamic–pituitary–gonadal axis activity. FSH values ranged from 0.70 to 42.65 IU/L, with a mean of 21.70 IU/L. Mean blood albumin was 4.07 g/dL, within normal range. SHBG levels ranged widely (5.69–147.00 nmol/L), suggesting differences in testosterone bioavailability. The mean SHBG was 59.23 nmol/L. Table 3 presents the summary statistics for this subgroup.

**Figure 2:**
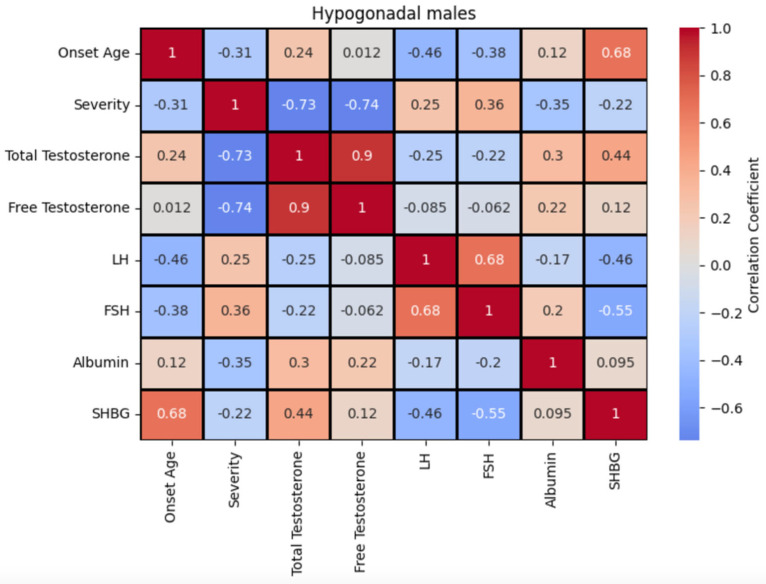
Correlation matrix between biomarkers in male patients with hypogonadism (correlation coefficient has no units). The highest positive correlation is between total testosterone and free testosterone (0.9), FSH and LH (0.68), and total testosterone and SHBG (0.44). Negative weak correlation between FSH and SHBG (−0.55) and LH and SHBG (−0.46). Correlation values might be influenced by the scarcity of data. Onset age is positively correlated to SHBG (0.68), and severity is negatively correlated to total testosterone (−0.73) and free testosterone (−0.74).

The correlation matrix between these biomarkers for the population with hypogonadism is presented in Figure 2. We also analyzed the correlation among these biomarkers, the onset age, and the severity provided by medical examination.

Semen analysis revealed azoospermia in 12 patients (Table 4). Among the remaining five, two had oligozoospermia, one had isolated teratozoospermia, one had asthenozoospermia, and one showed combined oligoasthenoteratozoospermia. Testicular biopsies were performed in five patients, revealing seminiferous tubule hyalinization, Sertoli cell predominance, and reduced Leydig cells.

#### 3.1.3. Population Without Hypogonadism

Table 5 shows the main biomarkers statistics in this group. This group demonstrated consistently higher androgen levels compared to the hypogonadal subgroup. The mean total testosterone level was 18.08 nmol/L, notably higher than the 6.36 nmol/L observed in the hypogonadism group. Free testosterone averaged 0.234 nmol/L, also significantly elevated relative to the hypogonadal mean of 0.091 nmol/L. Luteinizing hormone (LH) levels averaged 7.35 IU/L (range, 2.8–14.2), which was lower than the mean of 14.37 IU/L in the hypogonadism group, indicating distinct hypothalamic–pituitary–gonadal axis activity. FSH values ranged from 2.39 to 20.30 IU/L, with a mean of 8.10 IU/L. Mean serum albumin was 4.63 g/dL, slightly higher than the 4.07 g/dL found in the hypogonadal group. The mean SHBG level was 61.93 nmol/L, only slightly greater than the 59.23 nmol/L observed in the hypogonadism group.

**Figure 3:**
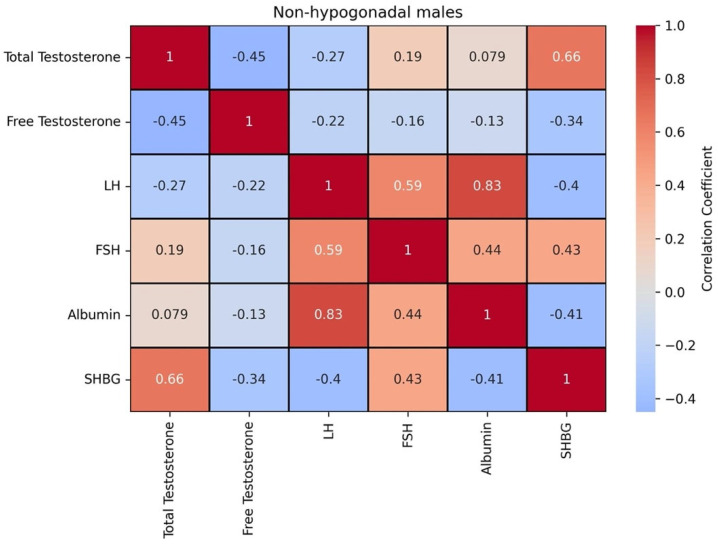
Correlation matrix between biomarkers in male patients without hypogonadism.

Figure 3 shows the correlation matrix between these biomarkers for the population without hypogonadism.

#### 3.1.4. Analysis of Statistical Differences Between Distributions

Table 6 shows the comparison of hormonal and biochemical markers between patients with and without hypogonadism and the result of the T-test performed on these biomarkers, ordered by statistical significance. The T-value measures how different the group means are relative to the variation in the data. A larger absolute t-value suggests a bigger difference between the groups. The *p*-value measures the statistical difference between the biomarkers in both groups; that is, is the difference between the two distributions of these biomarkers likely due to random chance, or is it statistically meaningful? Small *p*-values imply that the Null hypothesis (the samples come from the same distribution) should be rejected.

#### 3.1.5. Comparison of Hormonal Profiles

As can be observed in Table 6, all the biomarkers except SHBG show a significant difference between both groups, with free testosterone and total testosterone being the most significant. Differences in albumin, FSH, and LH are also statistically different at a level of 0.05. The group without hypogonadism exhibited significantly higher levels of total and free testosterone compared to those with hypogonadism. Luteinizing hormone (LH) and follicle-stimulating hormone (FSH) levels were markedly elevated in the hypogonadism group, consistent with hypergonadotropic hypogonadism and impaired negative feedback regulation of the hypothalamic–pituitary–gonadal axis. These findings contrast with the eugonadal group, where lower LH and FSH values reflect preserved endocrine feedback. While albumin and SHBG levels were slightly higher in the eugonadal group, these differences were minor and unlikely to meaningfully influence testosterone bioavailability. Notably, testicular volume was less than 12 mL in all the patients with hypogonadism, supporting the diagnosis of primary testicular failure.

#### 3.1.6. Biomarker Correlations

In the non-hypogonadism group, total testosterone and SHBG exhibited a strong positive correlation, while free testosterone and SHBG demonstrated a moderate negative correlation. This may reflect distinct regulatory mechanisms for bound versus free forms of testosterone. LH and albumin were also strongly positively correlated, suggesting potential shared regulatory influences. FSH is positively correlated with all the biomarkers, except for free testosterone. These findings, along with the moderate inverse relationship between SHBG and free testosterone, underscore the relevance of SHBG in modulating testosterone bioavailability (Figure 3). The correlation between these biomarkers with the onset age and the severity shows that the former is positively correlated to SHBG (0.68) and the latter is negatively correlated to total testosterone (−0.73) and free testosterone (−0.74).

In contrast, within the hypogonadism group, the strongest correlations were observed between total testosterone and free testosterone (0.9), between LH and FSH (0.68), and total testosterone and SHBG (0.44). SHBG displayed negative weak correlations with FSH (−0.55) and LH (−0.46). Albumin showed weak positive correlations with both total and free testosterone but was not strongly associated with other variables (Figure 2).

These findings might obviously be affected by data scarcity and should be interpreted with caution. Nevertheless, these correlations seem to be very logical.

### 3.2. Female Patients

All 20 female patients experienced menstrual irregularities requiring hormonal therapy. The mean age at menarche was 14 years (SD, 1.57). Four patients underwent menopause at a mean age of 35 years (SD, 5.56), with the earliest case occurring at 27 years. Only one woman had given birth at the age of 25. Menstrual disturbances were common, and 20% of the cohort experienced premature menopause. Four women had low anti-Müllerian hormone (AMH) levels (≤0.53 ng/mL). The mean AMH level was 0.30 ng/mL (SD, 0.21), and the median was 0.35 ng/mL. The average age of participants was 23 years (SD, 4.32), with a median age of 24 years.

Notably, the patient with the highest AMH level (0.5 ng/mL) was 17 years old, while the lowest level (0.01 ng/mL) was recorded in a 29-year-old woman. Ultrasound showed ovarian atrophy in three cases and a menopausal-appearing uterus in two of them. Remarkably, the woman with the lowest AMH level achieved embryo implantation via oocyte donation after 14 months of hormonal therapy to reach appropriate endometrial thickness. These findings indicate a predisposition to hypogonadism or earlier ovarian dysfunction compared to the general population (Table 7).

## 4. Discussion

Our study reveals a strikingly high prevalence of gonadal dysfunction in patients with Wolfram syndrome (WS), particularly among males. Hypogonadism was detected in 76.0% of male participants, with 89.5% of these cases classified as hypergonadotropic, characterized by low testosterone levels and elevated LH and FSH. These hormonal profiles, together with consistent histological findings—such as seminiferous tubule hyalinization, Sertoli cell predominance, and Leydig cell depletion—strongly suggest that primary testicular failure is the dominant pathophysiological mechanism, in agreement with previous studies [5,9,12,13].

Reproductive function was severely impaired: 70.6% of hypogonadal males had azoospermia, while the rest exhibited oligozoospermia, teratozoospermia, or asthenozoospermia. This degree of spermatogenic failure reinforces the importance of early fertility assessment and individualized reproductive counseling in WS males. Interestingly, in the subgroup without hypogonadism, total testosterone averaged 18.08 nmol/L versus 6.36 nmol/L in hypogonadal patients, while LH levels were significantly lower (7.35 vs. 14.37 IU/L), supporting an intact hypothalamic–pituitary–gonadal axis in the former group.

In contrast, the female cohort presents a more nuanced picture. All 20 women experienced menstrual irregularities, and 20% had premature menopause. Although hormonal contraceptive use may obscure clinical features of ovarian insufficiency, our findings—such as AMH levels ≤ 0.53 ng/mL in four patients, ovarian atrophy in three, and a postmenopausal uterus in two—indicate a reduced ovarian reserve. Notably, one patient with an AMH of 0.01 ng/mL and ultrasound evidence of atrophy achieved a pregnancy with donor oocytes after 14 months of hormonal therapy, highlighting the complex reproductive challenges in this group [19,20].

Our correlation analyses further support the existence of divergent endocrine profiles between hypogonadal and non-hypogonadal patients. In non-hypogonadal males, testosterone showed a strong positive correlation with SHBG, while free testosterone had a moderate inverse correlation with SHBG—pointing to different regulatory dynamics of free and bound androgens. Meanwhile, in the hypogonadal group, LH displayed weak correlations with other markers, suggesting reduced feedback responsiveness or a disrupted axis. The onset age is positively correlated with SHBG. Conversely, the severity is negatively correlated with the total testosterone and free testosterone. Both results are interesting as they show ways to delay the onset age of hypogonadism and reduce its severity through appropriate hormonal treatments.

Psychosocially, gonadal dysfunction has a tangible impact on quality of life. Reports of erectile dysfunction and relational difficulties among hypogonadal males illustrate the broader implications beyond biochemical markers. Although current guidelines, such as the PNDS and DIDMOAD, provide valuable frameworks [1,3], none include systematic gonadal evaluation, despite its significant physical, emotional, and reproductive consequences.

Finally, given WS’s genetic basis, it is imperative to implement early screening and genetic counseling protocols. Hormonal assessment (total and free testosterone, LH, FSH, inhibin B) and semen analysis should begin by age 14 in males, particularly if puberty is delayed or incomplete. In females, early AMH evaluation, even in the context of apparently normal menstrual cycles, may uncover early ovarian insufficiency [19,20]. Fertility preservation strategies and psychological support must also be considered as part of comprehensive care. To date, only one article has specifically addressed genetic counseling in WS, led by the principal investigator of this study [23], emphasizing the urgency of advancing research and clinical guidance in this area.

## 5. Conclusions

This study confirms that gonadal dysfunction is a prevalent and clinically significant feature of Wolfram syndrome (WS), predominantly manifesting as hypergonadotropic hypogonadism. Among males, 19 of 25 patients (76.0%) met diagnostic criteria for hypogonadism, including 12 cases of azoospermia and 2 cases of oligozoospermia. The mean total testosterone level in affected males was 6.36 ng/dL, substantially lower than the 18.08 ng/dL observed in the non-hypogonadal group. In females, 20% experienced premature menopause, and four individuals had AMH levels ≤ 0.5 ng/mL, with ovarian atrophy identified in three of these cases. These findings support primary gonadal failure as the predominant mechanism, although two patients exhibited hypogonadotropic hypogonadism, suggesting hypothalamic–pituitary axis involvement. Both cases of hypogonadotropic hypogonadism occurred in patients with early-onset insulin-dependent diabetes mellitus, as typically seen in WS. However, at the time of hormonal assessment, neither patient exhibited clinical signs of poor glycemic control (e.g., sustained hyperglycemia or ketoacidosis), nor were there indications of confounding factors such as significant weight loss, hyperprolactinemia, chronic illness, or neurological disease. These observations support the hypothesis that hypogonadotropic hypogonadism in these cases is likely intrinsic to the WS spectrum, rather than a secondary or functional phenomenon.

The high frequency of gonadal impairment—especially primary testicular failure in males and diminished ovarian reserve in females—highlights the urgent need to implement standardized protocols for gonadal function evaluation. Hormonal assessment (including testosterone, LH, FSH, and inhibin B) and semen analysis in boys from age 14, as well as AMH measurement and pelvic imaging in girls during early adolescence, should be incorporated into routine care. Given the profound psychosocial and reproductive consequences of hypogonadism, comprehensive care models must also include psychological support and tailored reproductive counseling. Furthermore, the hereditary nature of WS underscores the importance of structured genetic counseling. Future research should prioritize fertility preservation strategies to optimize long-term reproductive health and improve quality of life for individuals with WS.

## Figures and Tables

**Table 1:** Diagnostic criteria for males with hypogonadism.

Type	Total Testosterone	LH/FSH Levels and Clinical Features
Primary (Hypergonadotropic)	<300 ng/dL	Elevated LH/FSH. Testicular failure
Secondary (Hypogonadotropic)	<300 ng/dL	Low/normal LH/FSH. Hypothalamic/pituitary dysfunction

Source: Table created by the authors based on established clinical diagnostic criteria.

**Table 2:** General statistics in the male population. Hormonal and biochemical percentile values, mean, standard deviation, and range (min, max).

Statistic/Marker	Total Testos. (nmol/L)	Free Testos. (nmol/L)	LH (IU/mL)	FSH (IU/mL)	Albumin (g/dL)	SHBG (nmol/L)
*mean*	9.88	0.135	12.40	18.44	4.21	59.88
*sd*	7.06	0.079	6.91	13.50	0.52	26.95
*min*	2.76	0.026	1.20	0.70	2.90	5.69
*25%*	3.80	0.071	7.70	4.30	3.80	43.20
*50%*	5.99	0.106	11.45	20.30	4.20	55.00
*75%*	16.00	0.196	18.83	28.10	4.60	72.90
*max*	23.88	0.293	25.00	42.65	4.81	147.00

**Table 3:** Statistics in the hypogonadism group.

Statistic/Marker	Onset Age	Total Testos. (nmol/L)	Free Testos. (nmol/L)	LH (IU/mL)	FSH (IU/mL)	Albumin (g/dL)	SHBG (nmol/L)
*mean*	18.35	7.29	0.103	13.99	21.70	4.07	59.23
*sd*	3.60	5.43	0.059	6.83	13.52	0.53	29.27
*min*	15.0	2.76	0.026	1.20	0.70	2.90	5.69
*25%*	16.0	3.65	0.064	10.08	11.55	3.80	42.15
*50%*	17.0	4.22	0.077	13.53	25.40	4.10	51.00
*75%*	19.0	11.09	0.132	20.50	32.43	4.38	72.95
*max*	29.0	19.06	0.239	25.00	42.65	4.81	147.00

**Table 4:** Sperm analysis in hypogonadal male patients.

Sperm Analysis	Number of Patients	Percentage (%)
*Azoospermia*	12	70.6
*Oligozoospermia*	2	11.8
*Teratozoospermia*	1	5.9
*Asthenozoospermia*	1	5.9
*Oligoasthenoteratozoospermia*	1	5.9

**Table 5:** Statistics in the non-hypogonadism group. In this case, the onset age column is absent.

Statistic/Marker	Total Testos. (nmol/L)	Free Testos. (nmol/L)	LH (IU/mL)	FSH (IU/mL)	Albumin (g/dL)	SHBG (nmol/L)
*mean*	18.08	0.234	7.35	8.10	4.63	61.93
*sd*	5.17	0.043	4.39	6.98	0.14	19.85
*min*	9.64	0.182	2.80	2.39	4.48	38.80
*25%*	16.25	0.199	4.14	3.18	4.53	55.50
*50%*	18.10	0.242	6.62	5.42	4.60	58.50
*75%*	21.88	0.257	9.59	10.84	4.75	61.50
*max*	23.88	0.293	14.20	20.30	4.80	98.80

**Table 6:** Comparison of biomarkers according to hypogonadism status. Biomarkers are ordered by statistical significance. Boldface indicates the highest mean values for these biomarkers in both groups. Standard deviations of the biomarkers are provided within the brackets.

Biomarker	Hypogonadism Group (n = 19)	Non-Hypogonadism Group (n = 6)	T-Value	*p*-Value	Interpretation
*Free Testosterone*	0.103 (0.059)	**0.234** (0.043)	*−4.94*	*0.000053*	Extremely significant
*Total Testosterone*	7.29 (5.42)	**18.08** (5.16)	*−4.28*	*0.000275*	Highly significant
*Albumin*	4.07 (0.52)	**4.63** (0.14)	*−2.52*	*0.019120*	Significant
*FSH*	**21.70** (13.51)	8.10 (6.98)	*2.34*	*0.028102*	Significant
*LH*	**13.99** (6.86)	7.35 (4.38)	*2.21*	*0.036992*	Significant
*SHBG*	59.23 (29.27)	**61.93** (19.85)	*−0.21*	*0.835634*	Not Significant

**Table 7:** Female hormonal and reproductive findings.

Patient ID	Age (Years)	AMH (ng/mL)	Ovarian Atrophy	Menopausal Uterus	Pregnancy
*W1*	29	0.01	Yes	Yes	egg donor
*W2*	35	0.2	Yes	No	No
*W3*	28	0.4	Yes	Yes	No
*W4*	27	0.5	No	No	No

## Data Availability

The data that support the findings of this study are available from the corresponding author upon reasonable request.
